# Enzyme-activated probes in optical imaging: a focus on atherosclerosis

**DOI:** 10.1039/d1dt02198b

**Published:** 2021-09-27

**Authors:** Edward R. H. Walter, Saul M. Cooper, Joseph J. Boyle, Nicholas J. Long

**Affiliations:** Department of Chemistry, Imperial College London, Molecular Sciences Research Hub White City Campus Wood Lane London W12 0BZ UK n.long@imperial.ac.uk; National Heart and Lung Institute, Imperial College London London W12 0NN UK

## Abstract

Enzyme-activated probes enable complex biological processes to be studied in real-time. A wide range of enzymes are modulated in diseases, including cancer, inflammatory diseases and cardiovascular disease, and have the potential to act as vital diagnostic and prognostic biomarkers to monitor and report on disease progression. In this perspective article, we discuss suitable design characteristics of enzyme-activated fluorescent probes for *ex vivo* and *in vivo* optical imaging applications. With a particular focus on atherosclerosis imaging, we highlight recent approaches to report on the activity of cathepsins (K and B), matrix metalloproteinases (MMP-2 and MMP-9), thrombin, heme oxygenase-1 (HO-1) and myeloperoxidase (MPO).

## Abstract 2

This perspective provides an overview of the design requirements and fluorogenic mechanisms of enzyme-activatable probes in optical imaging, focusing particularly on detecting enzymatic targets overexpressed in vulnerable atherosclerotic plaque.

## Graphical abstract

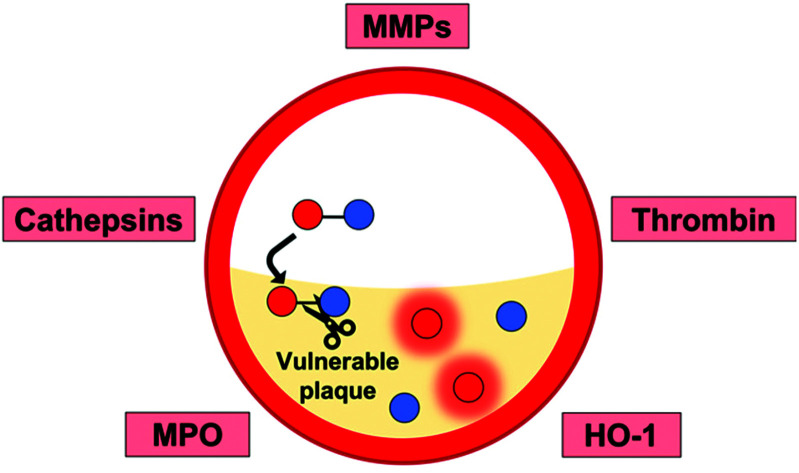

## 1. Introduction

Optical Imaging (OI) is a powerful, highly sensitive and cost effective non-invasive diagnostic technique routinely used in chemical biology.^1^ ‘Activity’ or ‘smart’ fluorescent probes are highly advantageous for use in OI, and typically display a rapid and selective fluorescence activation mechanism resulting in a high signal-to-noise ratio against the background signal. Additionally, such probes have the advantage of visualising direct molecular processes and abnormalities, compared to Molecular Resonance Imaging (MRI) and Ultrasound (US) which provide much more detailed anatomical information.

Over the years, a wide range of activity-based probes have been designed and developed to detect a number of metal ions,^2,3^ pH fluctuations^4^ and biologically relevant enzymes^5–7^ in real-time. In particular, enzymes represent important biomarkers for diagnosis and monitoring the progression of a number of chronic diseases such as Parkinson's disease,^8^ arthritis^9^ and many types of cancer.^10^ Therefore, enzyme-activated fluorescent probes are an important area in this field.

One growing area of interest is utilising enzyme-activated fluorescent probes for the OI of atherosclerosis. Acting as a prerequisite to Cardiovascular disease (CVD) and stroke – two leading causes of death worldwide,^11^ atherosclerosis is a chronic and often asymptomatic condition that is characterised by the narrowing of arteries following a steady build-up of plaque over a prolonged period of time.^12^ Atherosclerotic plaque broadly takes two phenotypes, named stable and vulnerable. One of the main challenges in this field is differentiating between these two dissimilar forms, particularly in the planning of medical interventions.

For example, vulnerable plaque is characterised by a high burden of inflammatory macrophages and a thin fibrous cap that is prone to rupture. One of the key processes in disruption is intraplaque hemorrhage (IPH), a bleed within the plaque itself, and strongly associated with more complete plaque disruption causing thrombosis and blockage of the lumen.^13,14^ Therefore, atherosclerosis is accelerated by IPH, and often results in the onset of ischemic stroke and myocardial infarction.^15^ In contrast, stable plaques display the opposite features and are significantly less prone to rupture.

In this perspective, we summarise the design aims and activation mechanisms that may guide the future development of enzyme-activated probes for disease diagnosis and prognosis. In particular, we focus on recently developed enzyme-activatable fluorescent probes for imaging atherosclerosis, and future directions that could be taken in this important area of fluorescence medical imaging. Chemical probes for other biological targets secreted by macrophages, such as cytokines, will not be discussed.

## 2. Enzyme-activated probes in OI

Enzyme-activated probes are highly advantageous for OI applications, allowing molecular processes to be detected in real-time. Probes of this nature typically contain an enzyme-specific functionality and a fluorescent reporter moiety to provide the signal output. During the design and development process, a number of different factors must be considered, including the photophysical properties of the fluorophore and biological considerations of the probe prior to, and following activation. Moreover, these design requirements for enzyme-activatable fluorescent probes are inherently generalisable so would also apply to other medical OI probes in various biochemical contexts.

### 2.1 Considerations in probe design

As with all fluorescent probes used in OI applications, the signal-transduction and photophysical properties of enzyme-activated fluorogenic probes must be carefully considered. The fluorescence readout observed following activation is most commonly intensity-based *via* a ‘turn-on/off’ mechanism. However, in some instances, the comparison of two or more wavelengths enables a ratiometric measurement. Such a ratiometric response is highly advantageous, due to enablement of self-calibration, eliminating artefacts resulting from the degree of cellular uptake or intracellular quenching during imaging.^16^ This facilitates much more precise assessment.

In a biological setting a low-energy excitation (long wavelength) is extremely desirable for two reasons. The first is reduced absorbance by endogenous biomolecules such as hemoglobin,^17^ allowing an increased depth of tissue penetration. To this end, a range of fluorophores with a NIR-1 (700–900 nm)^18^ and NIR-2 (1000–1700 nm)^19^ spectral window have been developed in recent years for *in vivo* imaging applications. Additionally, probes that enable two-photon excitation are also extremely sought-after, due to the use of the lower energy excitation of two individual NIR photons.^20^

It is also important to consider the relative energy difference between the absorbance and emission of light of a particular fluorophore – termed the Stokes’ shift. Fluorophores that display a large Stokes’ shift are preferred and prevent the self-absorption of emitted light by molecules in the ground state.^21^ Finally, the fluorescent reporter must also be photostable with a high quantum yield (*ϕ*) and large extinction coefficient (*ε*).

In addition to the photophysical properties of the fluorophore, a number of biological factors must also be considered. The probe must respond selectively to the necessary enzymatic target in a complex biological environment at physiological pH.^22^ Additionally, the probe, and the products generated following enzyme activation, must be non-cytotoxic in order to prevent intracellular damage during *in vivo* imaging applications.

In many instances, it is also desirable to incorporate biological targeting functionalities into probe design to ensure localisation in a specific region of the body or cellular compartment.^23^ The unique properties of functionalised nanoparticles are often used to this end, as a strategy for the delivery of small molecule-based probes. One such approach, used in several probes designed for activation by proteases, is the use of a peptide-appended pegylated graft co-polymer to aid delivery through enhanced circulation of the agent.^24–26^ Additionally, functionalised liposomal nanoparticles can encapsulate an OI probe for delivery to a specific vascular site.^27^ In a very interesting approach, the endogenous protective nanoparticle High-Density Lipoprotein (HDL) has been reverse engineered as a potential carrier for therapeutic and imaging reagents.^28,29^ Furthermore, inorganic nanoparticles (composed of iron oxide,^30,31^ gold^32^ or silica^33^) and solid lipid nanoparticles^34^ have been developed as a carrier for an array of imaging techniques.

A further approach, used to good effect in the application of lipophilic cation-functionalised probes to facilitate mitochondrial localisation, includes a small targeting group on the probe scaffold itself.^35,36^ An example by Urano and co-workers uses the specificity provided by antibodies to their cellular targets, in the application of activatable probe–antibody conjugates to cancer cells.^37^

The nature of the fluorophore itself can strongly influence the pharmacokinetic profile of the imaging agent, particularly in the case of extended systems for signal generation in high molecular weight probes. Therefore, an optimal balance between the desired NIR properties of a probe, and the undesirable perturbation it induces in the pharmacokinetics of a delivery system needs to be established. As such, strategies to improve localisation of an OI probe at the site of interest, through additional targeting moieties, encapsulation, or extended circulation times, are of great contemporary interest.^38^

### 2.2 Fluorescence activation mechanisms

**Fig. 1:**
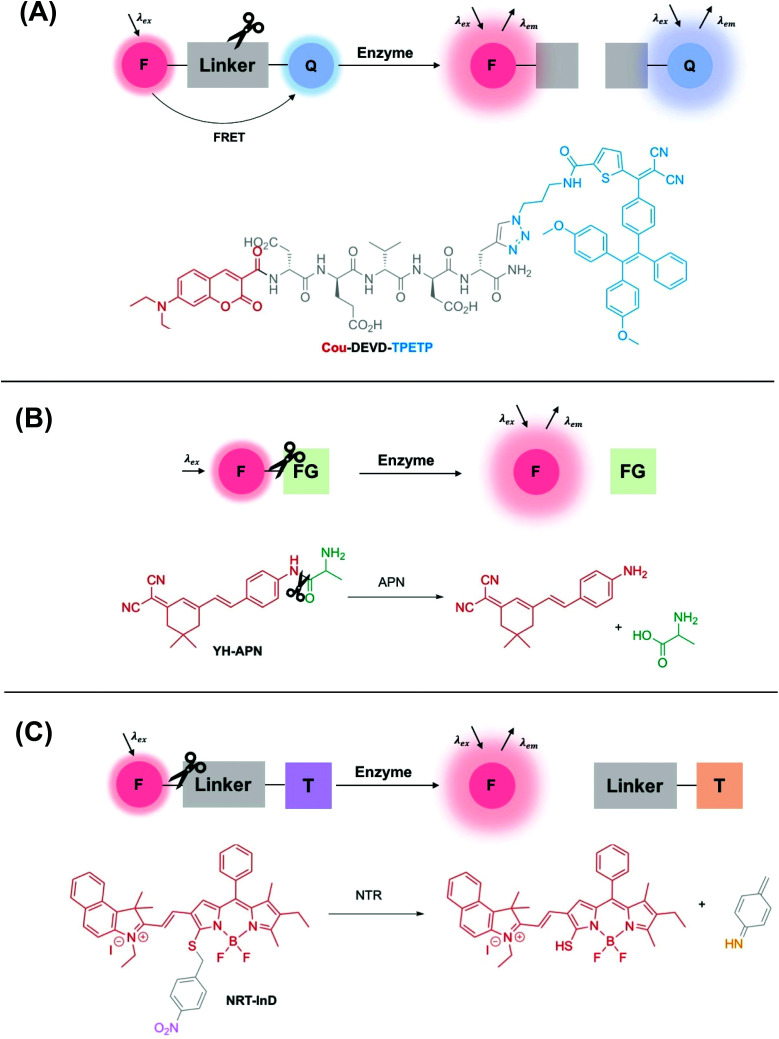
Schematic showing three common examples of enzyme-activity probes in the literature. (A) A ‘break-apart’ probe with a cleavable linker between two fluorophores *e.g.***Cou-DEVD-TPETP** from Liu and co-workers.^39^ Another common approach utilises self-quenching of two fluorophores linked by an enzyme cleavable peptide sequence. (B) An enzyme-specific functional group cleavage *e.g.***YH-APN** developed by Peng and Yoon,^40^ and (C) a self-immolative probe for nitroreductase, **NRT-InD** developed by Zhao and co-workers.^41^ F = fluorophore, Q = FRET pair/fluorescent quencher, FG = enzyme-selective functional group, T = trigger group.

A wide range of different fluorescence activation mechanisms in OI have been reported to date, in part reflecting a variety of different applications and enzymatic targets. Here, we summarise three common fluorescence activation mechanisms, with recently developed examples from the literature. The first two examples described in this section are particularly widespread and are characterised by an enzyme-specific cleavage that is accompanied by a fluorescence response. Such a cleavage is typically either a linker between two fluorophores (*e.g.* a specific, short peptide sequence) (Fig. 1A), or a functional group cleavage on a single fluorophore (*e.g.* an ester/thioester) (Fig. 1B).

Additionally, another common class of enzyme-activated fluorogenic probes involve the use of a self-immolative linker that masks the fluorescence of a fluorophore prior to enzymatic activation in the cellular environment. In this case, following enzyme activation, a chemical trigger group initiates a cascade of chemical reactions, initiating an increase in a fluorescence output (Fig. 1C).

Of course, other activation mechanisms are entirely possible too, but are not within the scope of this perspective. We point to two informative recent reviews in this area for a more detailed literature evaluation of a number of disease-relevant enzymes, particularly in oncology.^42,43^

The incorporation of a short and specific enzyme-cleavable peptide sequence that also acts as a linker between a pair of fluorophores is frequently the method of choice for monitoring the activity of a range of different proteases, a hydrolase enzyme, in real-time.^44–46^ Following selective enzymatic scission, a change in fluorescence response is observed, often due to the perturbation of Fluorescence Resonance Energy Transfer (FRET)††FRET is a distance–dependent interaction that involves the donation of energy from a donor to an acceptor fluorophore proving that there is a good spectral overlap, and there are in close proximity. between two fluorophores (Fig. 1A). Typically, to minimise the interference of a background signal, the acceptor fluorophore is deployed as a fluorescent quencher.

Liu and co-workers used this approach to design a dual-signal ‘turn-on’ probe for caspase-3 – an enzyme responsible for the initiation and activation of cell apoptosis. Probe **Cou-DEVD-TPETP** contains a coumarin donor fluorophore and a tetraphenylethenethiophene (TPETP) acceptor acting as a fluorescent quencher (Fig. 1A).^39^ Following caspase-3 initiated cleavage of the DEVD peptide, FRET was inhibited and accompanied by a ‘turn-on’ in coumarin fluorescence. Additionally, self-validation of caspase-3 activity was provided by TPETP, displaying a red fluorescence ‘turn-on’ due to aggregation of the released TPETP acceptor *via* aggregation-induced emission (AIE). Due to this dual-signal approach, **Cou-DEVD-TPET** was used successfully to self-validate caspase-3 activity in live fluorescence imaging of HeLa and MDA-MB-231 cells.^39^

It is also important to note that in some cases, fluorescence quenching can occur by photoinduced electron transfer (PET)‡‡PET is a non-radiative decay process that quenches luminescence. It is characterised by an electron transfer from the highest occupied molecular orbital (HOMO) of a receptor (electron donor) to the HOMO of a lumiphore in its excited state. prior to activation.^47,48^ A self-quenching mechanism can also be utilised to good effect with two fluorophores in close proximity, quenching the fluorescence prior to activation. Probes with these design features are collectively named enzyme “break-apart” probes, with enzymatic cleavage accompanied by a fluorogenic response.

Probes for other types of hydrolase enzymes, such as esterases and thioesterases, contain enzyme-cleavable ester and thioester functional groups respectively.^49–51^ A similar functional group cleavage activation mechanism is observed for aminopeptidases, and this approach was used by Peng and Yoon to develop a CD13/aminopeptidases (APN) N-activatable fluorescent probe for tracking metastatic cancer (Fig. 1B). Probe **YH-APN** comprises a dicyanoisophorone fluorophore and an APN selective recognition site, l-alanine. Following a selective enzymatic activation by APN, **YH-APN** displayed a large Stokes’ shift of 205 nm and a detection limit of 0.13 ng mL^−1^.^40^ Crucially, from cell morphology, **YH-APN** could distinguish cells in a mixed cultivation of normal (LO2) and cancerous cells (Hep-G2), an experiment designed to simulate the environment of a tumour.^52^ To achieve the mixed cultivation, cell planting and culture was achieved in a step-wise manner, within the same confocal dish over a 48 h period. A fluorescence signal of 655–755 nm was detected in the mixed culture and attributed to enzyme activation of **YH-APN** in Hep-G2 cells only. Furthermore, in the study, cancerous tissues were successfully identified by *in situ* spraying, outlining a high tumour-to-normal tissue ratio. Lesions smaller than 1 mm could be imaged precisely, superior to other diagnostic techniques used in clinical practice such as MRI and US. Such approaches, therefore, show a great deal of promise for use in image-guided surgery in the future.^40^

Finally, ‘turn-on’ fluorescent probes incorporating a self-immolative linker are increasingly being developed to detect disease-relevant enzymes. For example, in 2019 Zhao and co-workers developed a series of BODIPY-based activity probes for nitroreductase (NTR) and NAD(P)H:quinone oxidoreductase isozyme 1 (NQO1) for imaging hypoxia in cancer cells (Fig. 1C).^41^ All probes in this study contained a self-immolative benzyl thioether linker, that resulted in a bathochromic-shift in the absorbance and emission wavelength maxima following activation by the enzymatic target. For example, following detection of NTR (in DMSO/Tris-HCl, v/v 2 : 8), probe **NTR-InD** displayed a significant decrease in fluorescence at 612 nm (*λ*_ex_ = 535 nm) and a 12-fold ‘turn-on’ in NIR fluorescence at 900 nm (*λ*_ex_ = 730 nm). A highly favourable Stokes’ shift of 170 nm was displayed. Such a significant bathochromic-shift in fluorescence following enzyme activation enabled **NTR-InD** to be analysed in A549 cells by two-channel ratiometric fluorescence imaging. Additionally, promising results were displayed in a murine *in vivo* model where a time-dependent NIR fluorescence was displayed specifically in the tumour.^41^

## 3. Enzyme-activated probes for atherosclerosis imaging

Over the years an array of invasive and non-invasive medical imaging techniques has been used to detect the pathogenesis of atherosclerosis. Non-invasive imaging modalities utilising an assortment of biological targeting moieties for use in MRI,^31,53,54^ OI,^55^ Photoacoustic Imaging (PAI)^56^ and Positron Emission Tomography (PET)^57,58^ have been developed, and summarised in an informative and recent review by Gao and co-workers.^59^

Here, in contrast, we summarise recent efforts to develop *activatable fluorescent probes* for enzymatic biomarkers associated with the diagnosis and pathogenesis of atherosclerosis. The primary challenge in this field is accurately differentiating between stable and vulnerable plaque, the latter of which is significantly more prone to rupture, due in part to the high burden of inflammatory macrophages.

Macrophages are responsible for the secretion of enzymes, reactive oxygen species (ROS) and cytokines as the primary response of a host to disease.^60,61^ Consequently, the development of chemical probes to monitor the enzymatic activity of macrophages is a popular approach to image plaque instability in atherosclerotic lesions.^62^ These include recent approaches to report on the activity of proteases (cathepsins, matrix metalloproteinases (MMPs), and thrombin), heme oxygenase-1 (HO-1) and myeloperoxidase (MPO). Due to commonality of molecular disease mechanisms, the enzymatic biomarkers developed for atherosclerosis may also be useful markers of other diseases, characterised by perturbation of the same enzyme activity.^42,43,61^

### 3.1 Monitoring the enzyme-activity of proteases

As described in section 2, the design of enzyme-activatable probes for proteases predominantly involves the incorporation of a specific enzyme-cleavable peptide sequence as a linker between two fluorophores. Proteases (*e.g.* cathepsins, MMPs and thrombin) are a class of enzymes that are primarily responsible for the digestion of the extracellular matrix (ECM), the weakening of the fibrous cap, and subsequently leading to plaque destabilisation and rupture from this vulnerable state.^63,64^ Additionally, proteases play a major role in vascular remodelling, and over time can lead to the development of more advanced atherosclerotic plaques, and possible thrombus formation.

#### 3.1.1 Cathepsins

**Fig. 2:**
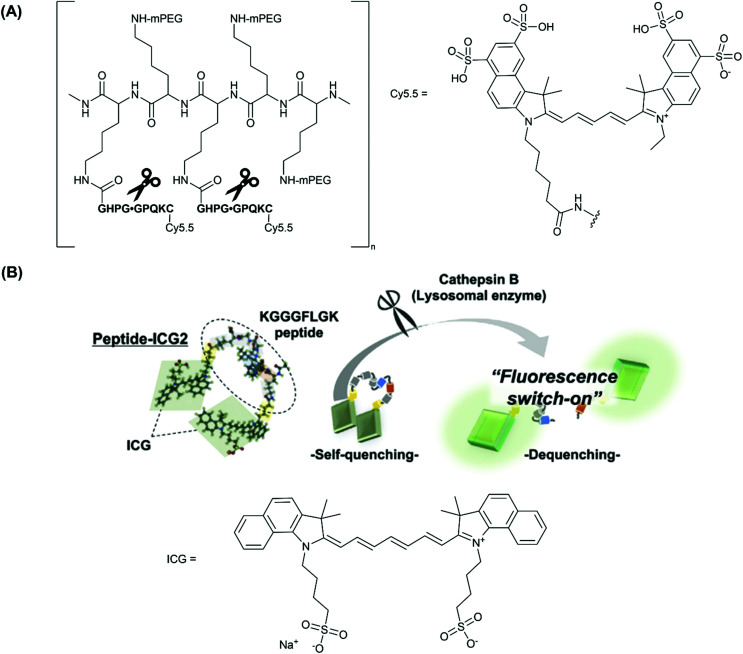
(A) The structure of **CatK-FITC-PGC-Cy5.5**, emphasising the three modular components; the Cy.5.5 unit, a CatK-specific GHPGGPQKC peptide substrate (cleaved at the glycyl-glycyl bond), and partially pegylated polylysine graft co-polymer to aid delivery of the agent, reported by Weissleder and co-workers.^26^ (B) The fluorescence ‘turn-on’ mechanism following enzymatic cleavage by cathepsin-B and the elimination of self-quenching utilised in **peptide-ICG2**, reported by Ogawa and co-workers.^27^ Reprinted with permission from ref. 27. Copyright (2019) Elsevier B.V.

Cathepsins are a member of the cathepsin family of cysteine proteases, and two members of this family, cathepsin K and B, have been shown to be upregulated in atherosclerotic lesions.^25,65^ Therefore, over the past decade a number of studies have been conducted in the development of new fluorescent enzyme-activatable “break-apart” probes for cathepsins. Most notably, Weissleder and co-workers have developed a NIR Cy5.5-labelled peptide co-polymer-based probe, **CatK-FITC-PGC-Cy5.5**, displaying an increase in fluorescence intensity following cathepsin K (CatK) specific activation in a murine model of atherosclerosis.^26^ CatK-initiated cleavage results in the separation of the fluorophores and amplification of the fluorescent signal intensity. A 3.5-fold increase in signal intensity was seen compared to a peptide-based control probe, and a 13-fold increase compared to PBS (measuring autofluorescence). The structure of the probe is depicted in Fig. 2A.

Earlier studies have also investigated a cathepsin B (CatB)-activated analogue in a murine atherosclerosis model.^24,25^ Indeed this probe, **ProSense750/VM110**, was used in the first demonstration of real-time intravascular Near-Infrared Fluorescence (NIRF) catheter-based imaging, by Jaffer and co-workers in 2008.^66^ Ultimately, two forms of commercially-available cathepsin-activated NIRF probes have been developed, based on the scaffold outlined above; **ProSense 750/VM110**, activated by cathepsins B, L and S, and **ProSense680**, activated by cathepsins B, L, S, K, V and D.^67^

More recently, Ogawa and co-workers employed another fluorescent ‘turn-on’ probe selective for CatB, designed to target macrophages in vulnerable atherosclerotic plaque, whilst avoiding the use of large co-polymer scaffold.^27^ Rather, phosphatidylserine (PS)-functionalised liposomes were used as a macrophage-specific delivery mechanism. In this study, indocyanine green (ICG, Fig. 2B), an FDA (U.S. Food and Drug Administration) approved NIR-1 cyanine dye, was used as the fluorophore of choice.

Probe **peptide-ICG2** contains two ICG fluorophores linked *via* a small peptide sequence (KGGGFLGK) that can be selectively cleaved by cathepsin B. Initially, the NIR emission of **peptide-ICG2** was quenched 15-fold compared to ICG, assumed by the authors to be due to π-stacking between the two ICG moieties (Fig. 2B). Following addition of cathepsin B, a time-dependent increase in the fluorescence intensity at 845 nm was observed in sodium acetate buffer (20 mM, pH = 5). Such an increase was attributed to the elimination of ICG self-quenching, and verification of cathepsin B-dependent peptide cleavage was confirmed following addition of leupeptin, a classical cathepsin B inhibitor.^68^

For *in vitro* and *ex vivo* imaging applications, **peptide-ICG2** was encapsulated in a (PS)-functionalised liposome to target macrophages that had infiltrated vulnerable atherosclerotic plaque.^69^ For NIR-1 dyes, encapsulation in an nanocarrier platform can be highly advantageous as it dramatically increases the circulation time of the probe, allowing substantial accumulation in a biological target of choice. It is also thought that the stability of ICG and other cyanine dyes can increase considerably following encapsulation.^70^

NIR *ex vivo* fluorescence imaging of the aortae of a murine imaging model (Apolipoprotein E knockout (ApoE^−/−^)) confirmed that the CatB-induced fluorescence signal observed was located in atherosclerotic plaque macrophages. Analogous results have been displayed in work by Kim and co-workers.^71^ In the latter, a weaker fluorescence intensity was displayed in mouse aortas with a high-fat diet following addition of atorvastatin, a known statin medication widely characterised to prevent cardiovascular disease by reducing plaque.^71^ Furthermore, it was suggested by the authors that a weaker fluorescence intensity correlates with more stable atherosclerotic plaque, an outcome that could not be predicted from a visual inspection.^71^ However, without any internal calibration or ratiometric response, this must be expressed with some caution as intensity-based probes can have systematic errors such as differing cell uptake, potentially affecting the intensity of the fluorescence output observed. One major limitation of self-quenching fluorophores is this inherent difficulty in obtaining ratiometric measurements. Such a response is achieved more readily when a donor–acceptor FRET quenching mechanism is employed.

Taken together these results are still very encouraging and demonstrate that OI probes for cathepsins are excellent candidates for imaging plaque vulnerability.

#### 3.1.2 Matrix metalloproteinases (MMPs)

Matrix metalloproteinases (MMPs) are a family of 23 zinc-dependent proteinases associated with various physiological and pathological process. Consequently, a large number of enzyme-activatable probes have been developed to detect MMP activity in fluorescence cancer imaging.^72–74^ Whilst MMP activity probes have been used to image and measure in atherosclerotic plaques, these are more limited.

**Fig. 3:**
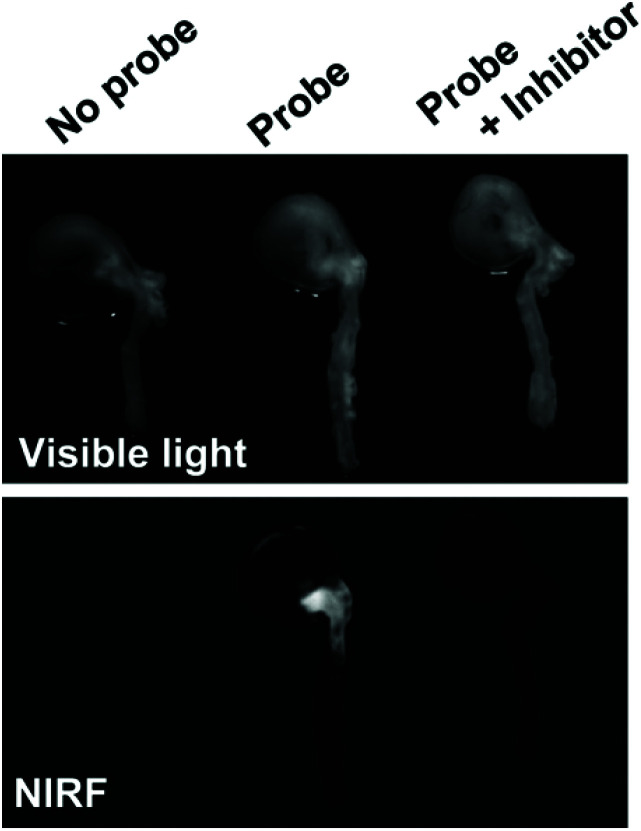
*Ex vivo* analysis of MMP-2 and MMP-9 activity in atherosclerotic aortas in a ApoE^−/−^ murine model by Deguchi and co-workers.^75^ Fluorogenic selectivity was confirmed following addition of MMP-2/MMP-9 inhibitor III, resulting in no significant increase in NIR fluorescence *versus* the control. Reprinted with permission from ref. 75. Copyright (2006), Wolters Kluwer Health.

In a notable example, Deguchi and co-workers developed a NIR Cy5.5-based self-quenching fluorogenic probe incorporating two Cy5.5 fluorophores linked *via* a GGPRQITAG peptide sequence on a pegylated polylysine backbone.^75^ The NIR probe was based on work by Huang and co-workers,^76^ and demonstrated a 200-fold increase in fluorescence intensity following cleavage by MMP-2 and MMP-9.^75^*Ex vivo* fluorescence imaging of atherosclerotic aortas of a ApoE^−/−^ murine model showed an MMP-2/MMP-9 specific increase in NIR fluorescence (Fig. 3). Furthermore, histological validation confirmed that NIR fluorescence was localised in macrophage-rich regions of the aorta. *In vivo* analysis revealed an enhanced MMP activity in atherosclerotic lesions, indicating that related probes could help distinguish stable and vulnerable plaques.^75^ However, as with **peptide-ICG2** (described in section 3.1.1), the non-ratiometric behaviour of these probes would limit precision of *in vivo* imaging applications.

#### 3.1.3 Thrombin

Thrombin is another serine protease with a significant role in a number of normal and pathological biological processes, including the development of vascular thrombosis. The enzyme is implicated in the pathophysiology of atherosclerosis in several ways,^77^ directing researchers to invent a number of probes to detect and report on its activity. For example, Tung and co-workers have developed a thrombin-activated molecular NIR probe based upon a similar co-polymer scaffold to that employed in the group's other published works for imaging macrophage protease activity.^78^

**Fig. 4:**
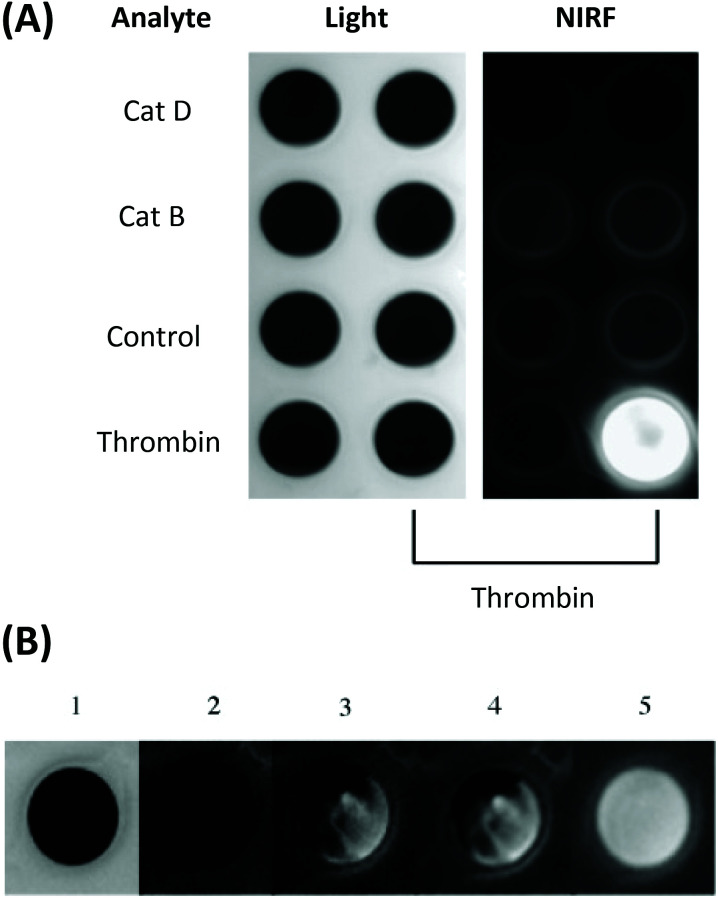
(A) The specificity of the thrombin-activated NIR fluorescent probe in PBS buffer (probe concentration = 0.5 μM). (B) Time-dependent increase in NIR fluorescence in whole blood with exogenous thrombin. 1 = light, 2 = NIR fluorescence image before the addition of thrombin, 3–5 = images at 3 min, 60 min, and 24 h respectively.^78^ Reprinted with permission from ref. 78. Copyright (2002) WILEY-VCH Verlag GmbH.

Another probe developed by Tung and co-workers bears a NIR Cy5.5-labelled peptide, containing a thrombin-activated cleavable peptide substrate, appended to a partially pegylated polylysine grafted co-polymer as a delivery vector. An average of 23 fluorophores were attached to each polymeric carrier molecule. A high specificity for thrombin was demonstrated in PBS buffer, with no NIR fluorescence observed without thrombin (control) or following addition of CatD or CatB (Fig. 4A). Additionally, such a probe was shown to exhibit an 18-fold increase in fluorescence intensity *in vitro*, using both exogenous thrombin in human blood (Fig. 4B) and in a mouse intravascular thrombosis model.^79^ In the latter case, the probe could be detected in the loci of the induced thrombi. Whereas these studies have shown the possibility of significant uptake of this probe in fresh thrombi, it has not been investigated in the context of older thrombi. Nevertheless, likewise to cathepsin and MMP-activated agents, thrombin-activated probes for OI are expected to be relevant in a wide range of disease contexts.

### 3.2 Heme oxygenase-1 (HO-1)

The vast majority of probes described in section 3.1 make use of a short, cleavable peptide sequence to facilitate enzymatic cleavage at sites of interest. However, a focus on different enzyme targets can facilitate activatable NIR probes based on alternative molecular scaffolds. Such scaffolds have the potential to be synthetically more accessible than high molecular weight bioconjugates based on co-polymers, peptides or proteins.

Heme oxygenase-1 (HO-1) plays a crucial role in vascular health, playing a protective role in hemorrhage and hematoma resolution.^80^ HO-1 is induced during the IPH of vulnerable plaque, and has somewhat been neglected as a biomarker in atherosclerosis imaging to date.

**Fig. 5:**
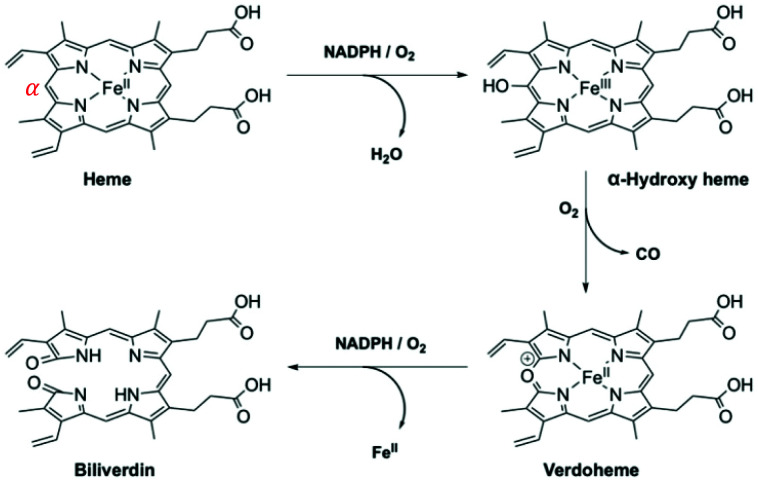
HO-1 catabolism of ‘free’ heme.^85,86^

HO-1 catalytically degrades ‘free’ (or unbound) heme, with regioselectivity for the α-*meso*-position, from which it releases carbon monoxide (CO) (Fig. 5).^81^ Whilst heme is an essential prosthetic group in numerous enzymes and hemoglobin, in the ‘free’ form it is cytotoxic due to the generation of highly reactive oxygen intermediates. During this three-step process bilirubin is formed, and ferrous iron is directed to safe storage in ferritin, preventing the generation of reactive oxygen species *via* the Fenton reaction.^82^ Each of the degradation products of heme/HO-1 (bilirubin, CO and Fe-ferritin) are well-known to display antioxidant and antiapoptotic properties.^83,84^

**Fig. 6:**
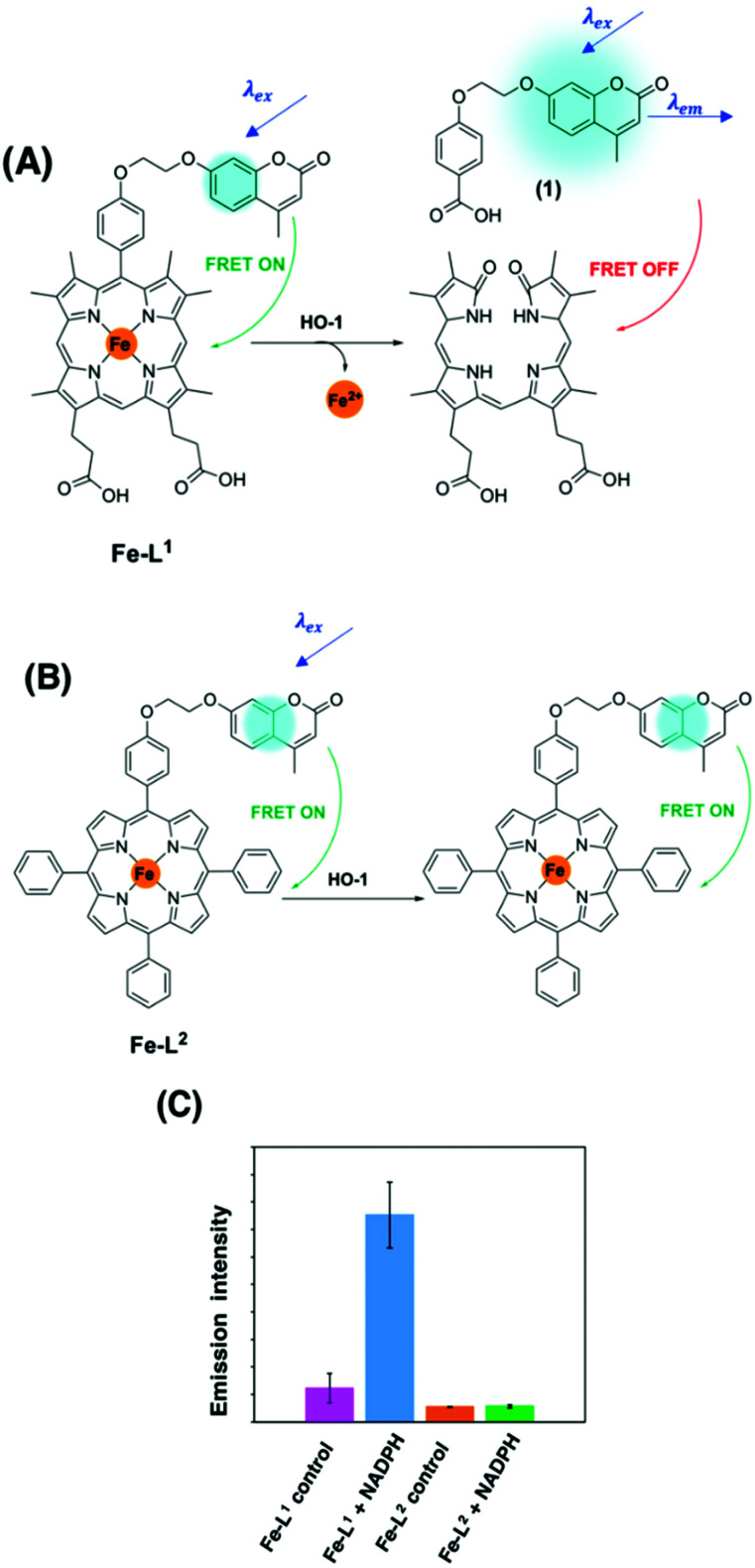
(A) HO-1 catalysed Regioselective α-cleavage of **Fe–L1**. (B) Diad **Fe–L2** and (C) the change in coumarin fluorescence at 383 nm of **Fe–L1** and **Fe–L2** in *E. coli* lysates with and without incubation with NADPH.^85^ Reprinted with permission from ref. 85, https://pubs.acs.org/doi/abs/10.1021/jacs.0c12864. Further permissions related to the material excerpted should be directed to the ACS.

To the best of our knowledge, there have been no known chemical probes to report on the activity of HO-1, holding back this field of research considerably. Our group has recently reported the design, development, photophysical and biological characterisation of the first known chemical probe to report on the activity of HO-1.^85^ Probe **Fe–L1** (Fig. 6A) is based on a symmetrical analogue of heme, known to display an activity for HO-1.^87^ The α-carbon atom was functionalised with a hydroxycoumarin donor fluorophore, and displayed an efficient FRET to the porphyrin acceptor moiety prior to HO-1 catabolism.^85^

HO-1 activity was quantified in *Escherichia coli* (*E. coli*) lysates overexpressing human HO-1 (hHO-1) in a proof-of-concept study. Following addition and incubation with NADPH, a *6-fold* increase in coumarin fluorescence intensity *versus* the control was reported. Mass spectrometry analysis (LC-MS and MALDI-MS) determined that porphyrin cleavage was regiospecific at the α-position, forming compound **1**, and perturbing the efficient FRET process from the coumarin donor to the porphyrin acceptor moiety (Fig. 6). Diad **Fe–L2** (Fig. 6B) was not a substrate for HO-1 and was subsequently used as a control.^85^

The development of a FRET break-apart probe for HO-1 is a significant step forward towards the translation of such an approach into a real-time imaging agent. Such an advancement would be a valuable addition to this field and could allow the detection of IPH and plaque vulnerability, as well as other internal hemorrhages caused by aneurysm or stroke. One key area to improve on in the near future is the development of new analogues with red-shifted excitation and emission wavelengths.

Currently, **Fe–L1** displays photophysical properties within the ultraviolet (UV) region of the electromagnetic spectrum. However, high energy excitation is undesirable in OI due to a poor biological tissue penetration and can trigger intracellular damage as a consequence of the formation of ROS. Work in our research group is currently on-going to develop analogues of **Fe–L1** with red-shifted absorbance and emission wavelengths for live-fluorescence imaging applications. For this purpose, probes with a high FRET efficiency remain essential, enabling a high signal-to-noise ratio and maximising the ‘turn-on’ increase in fluorescence observed following detection of HO-1 activity.

### 3.3 Myeloperoxidase (MPO)

Myeloperoxidase (MPO) is a member of the heme peroxidase family that catalyses the formation of a number of reactive oxidant species including hypochlorous acid and peroxynitrite. MPO utilises hydrogen peroxide (H_2_O_2_) and a chloride anion (Cl^−^) as substrates to form hypochlorous acid (HOCl).^88^ At physiological pH, approximately half of all HOCl exists as a hypochlorite anion (OCl^−^), an extremely strong natural oxidant capable of causing tissue damage during inflammation.

MPO is secreted by neutrophils and macrophages in human atherosclerotic lesions.^89,90^ Therefore, MPO provides another interesting enzymatic biomarker for the diagnosis and pathogenesis of atherosclerosis, through the detection of HOCl/OCl^−^ activity.

**Fig. 7:**
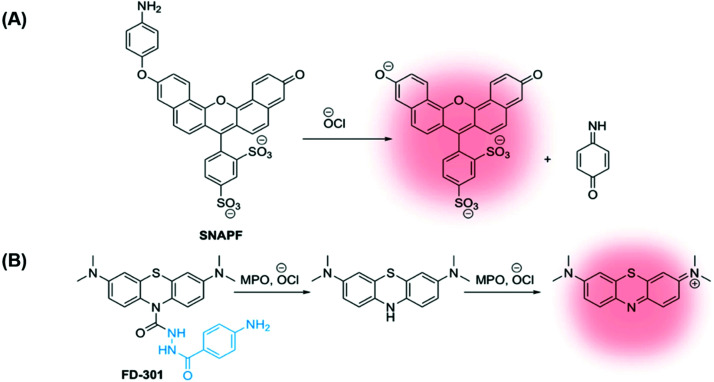
(A) Xanthene-based probe SNAPF for the detection of HOCl in atherosclerotic-associated macrophages, developed by Libby and co-workers.^91^ (B) A methylene blue (MB)-based NIR emissive ‘turn-on’ probe, **FD-301**, to detect basal MPO activity. MPO inhibitor 4-aminobenzoic acid hydrazide is highlighted in blue.^92^

In 2007, Libby and co-workers developed a self-immolative xanthene-based probe **SNAPF**, for selective detection of HOCl in MPO+ macrophages in atherosclerotic lesions. Probe **SNAPF** displayed an 8-fold increase in fluorescence intensity in PBS buffer at physiological pH (*λ*_ex_ = 625 nm, *λ*_em_ = 676 nm), with a high specificity over other reactive oxygen and nitrogen species (Fig. 7A).^91^ Additionally, **SNAPF** was able to detect the generation of HOCl in a murine *in vivo* model and in frozen tissue sections of human atherosclerotic plaque. Such a study showed great promise and was one of the first of its kind.^91^

More recently, Yi and Wei developed a NIR emissive ‘turn-on’ probe to detect basal MPO activity, **FD-301** (Fig. 7B).^92^ 4-Aminobenzoic acid hydrazide, a known MPO inhibitor,^93^ has been incorporated into the design of **FD-301**, facilitating binding to the MPO active site. However, from molecular docking and *in vitro* experiments, it was determined by the authors that the enzyme activity in **FD-301** is not inhibited. This is likely due to the steric hindrance resulting from the presence of the methylene blue (MB) fluorophore in the structure of the probe. Such an approach is advantageous enabling the MB fluorophore to be in close proximity to the MPO active site, reducing complications arising from the short half-life of HOCl. Following formation of HOCl, the hydrazine bond in **FD-301** is cleaved and a ‘turn-on’ increase in MB fluorescence is observed.^92^

The study by Yi and Wei shows promising data for the selective detection and quantification of HOCl and, by implication MPO activity *in vivo*. In this case, **FD-301** was studied *in vitro* and *in vivo* in murine models for arthritis and ulcerative colitis.^92^ However, using a similar probe design and imaging approach in the future could accurately detect the overexpression of MPO in macrophages found in human atherosclerotic lesions.

## 4. Summary and perspective

Using recent approaches from the literature, we have summarised the desirable design considerations and activation mechanisms for enzyme-activatable probes in OI applications. With a particular focus on atherosclerosis imaging, and the detection of vulnerable atherosclerotic plague, an array of relevant enzymatic biomarkers has been evaluated, encompassing proteases (cathepsins, MMPs and thrombin), HO-1 and MPO.

Differentiating between vulnerable and stable plaque remains the greatest challenge in this field. Whilst approaches described here are promising, they predominantly involve a “turn-on” activation mechanism, rather than a ratiometric behaviour. It remains key that new probes are developed in the future to ensure vascular diseases are detected earlier and more efficiently, before the onset of severe complications. The enzymes that are overexpressed in atherosclerosis also play key roles in a range of other chronic disorders, marked by inflammation and wound healing. Conversely, much information could be gained by repurposing enzyme-activated probes developed for other diseases, such as cancer. Such a strategy would improve the hugely challenging synthetic complexity which is often characteristic of enzyme-activatable probes. Ultimately, however, the development of new fluorogenic biomarkers for atherosclerosis is really required to significantly increase our understanding of this disease.

In particular, enzyme-activated fluorescent probes to image atherosclerotic lesions offer extremely promising applications in catheter-based OI. However, further clinical application of such probes is still dependent on the availability of a clinically approved catheter.^67^ In any case, the development of new fluorescent probes for less invasive *in vivo* imaging applications is also highly desirable. New NIR probes with high quantum yields, high stability, a high Stokes’ shift and low toxicity are required to this end. Furthermore, probes utilising two-photon excitation are also becoming increasingly desirable to facilitate greater depth penetration.

There are several challenges in developing OI agents applicable to atherosclerosis. These include a major trade-off between accessibility and wavelength. Longer wavelengths are better for tissue penetration and minimise tissue damage. For example, UV-excitable probes would be unacceptable. Yet, broadly, the longer the wavelength, the larger the dye molecule. Large fluorophores present challenges with entry in and out of the cells and tissue and may well present significant issues with steric hindrance of the enzyme site. Toxicity is a further consideration, particularly given the tissue is itself diseased. Appropriate instrumentation and route of delivery still remain challenges, although minimally invasive intracoronary approaches are now commonplace.

Other techniques, not discussed in detail over the course of this perspective, such as Photoacoustic Imaging (PAI) and luciferase-induced Bioluminescence Imaging (BLI), are appealing alternatives to OI. For example, PAI combines the advantages of OI and US imaging modalities, and possesses a higher intravascular resolution compared to MRI and CT,^94^ as well as a superior tissue penetration over OI.^95^ PAI is being validated at preclinical studies, where it allows relatively high-resolution imaging of vessels. Additionally, the technique is being incorporated into multimodality coronary imaging. Enzyme-activatable PAI probes have been developed for MMP-2,^56,96^ and will form a vital part of imaging toolkit to improve the diagnosis of vascular disease moving forward.

Luciferase-based imaging is already widely used in preclinical application, albeit predominantly based on genetic modifications, of a sort that would be challenging to translate clinically. For example, it has been widely used to image gene activation, based on insertion of artificial constructs comprised of gene regulatory sequence and an exogenous luciferase gene from firefly or jellyfish. A further evolution of this approach has been Bioluminescence Resonance Energy Transfer (BRET) which has primarily been used in the imaging of cell signalling.

Dual-modal imaging techniques are highly advantageous and will be further explored in the future. Such approaches have the potential to utilise an enzyme-activatable fluorophore to directly report on molecular abnormalities, in combination with a modality to report in detail on anatomical abnormalities. The combination of OI with MRI, for example, is one possibility due to the high spatial resolution and depth penetration that is characteristic of MRI, but both significantly lacking in OI. Research to this end is currently of major interest,^97,98^ and is likely to be studied further in the near future to develop the next generation of chemical probes for atherosclerosis imaging.

It is hoped that this perspective will provide an insight into the enzymatic targets for OI imaging of vulnerable atherosclerotic plaques, representing an exciting and emerging field within medical imaging. There are a number of directions to explore in the future, that could improve the diagnosis of vascular disease.

## Conflicts of interest

The authors have applied for IP protection for a novel class of HO-1 fluorescence probes.

## Supplementary Material
